# Supplementary material for: All-Atom MD Simulations of the HBV Capsid Complexed with AT130 Reveal Secondary and Tertiary Structural Changes and Mechanisms of Allostery
Source: Viruses. 2021 Mar 26;13(4):564. doi: 10.3390/v13040564 (PMC8065791; doi:10.3390/v13040564)
Supplement: Supplementary file 1 [file viruses-13-00564-s001.pdf]

Supplementary Materials: All-Atom MD Simulations of the HBV Capsid Complexed with AT130 Reveal Secondary and Tertiary Structural Changes and Mechanisms of Allostery

Carolina Pérez-Segura[1], Boon Chong Goh[2] and Jodi A. Hadden-Perilla[1,*]

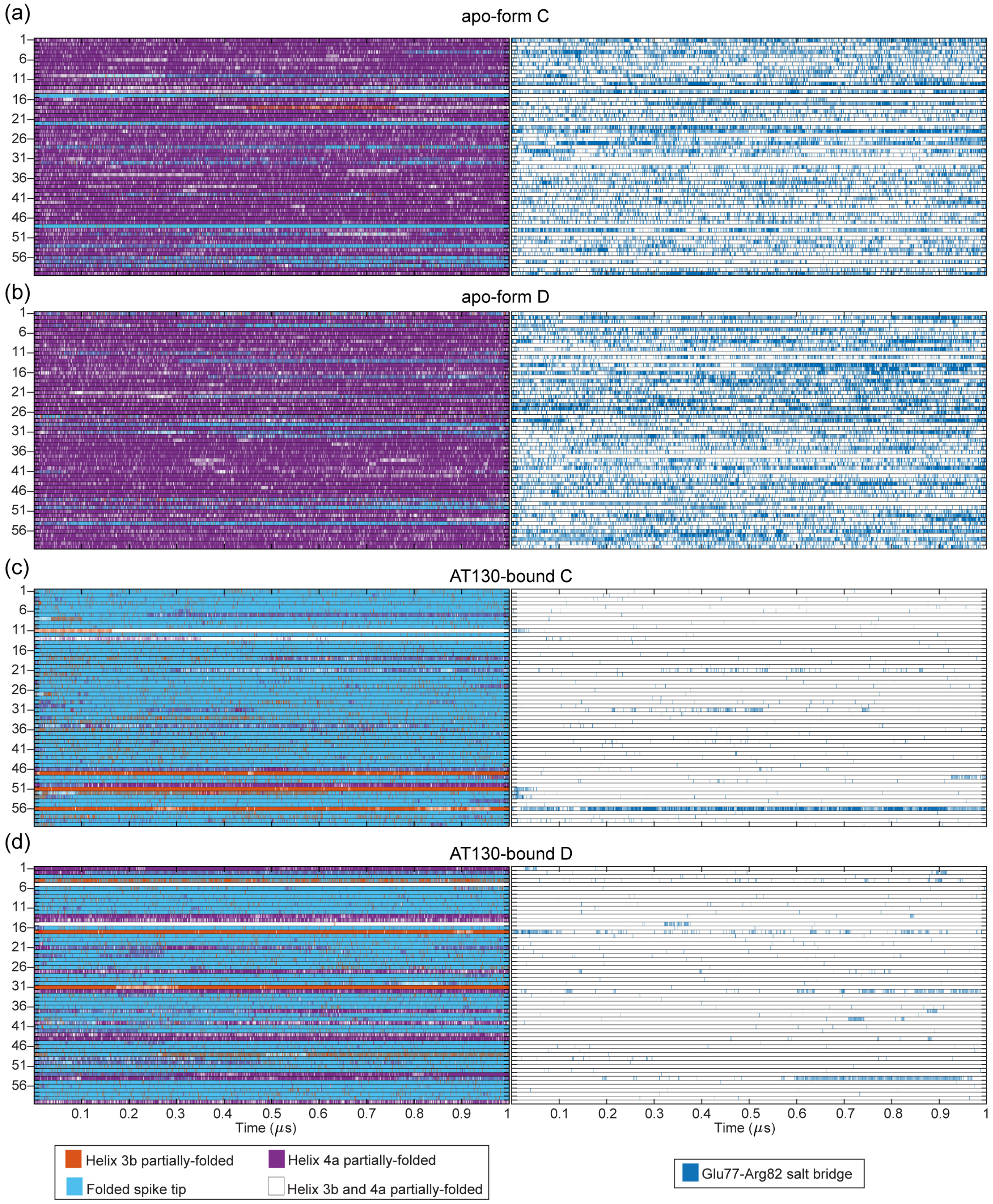

Figure S1. Spike helicity and Glu77–Arg82 salt bridge. Heat maps showing time series of spike tip helicity (left) and presence of Glu77–Arg82 salt bridge (right) for apo-form (**a-b**) and AT130-bound (**c-d**) CD dimers. Fully-folded spike tip is defined as having helix 3b intact through its C-terminus at Asn75 and helix 4a intact from its N-terminus at Pro79; less helical content is considered partially-folded. The Glu77–Arg82 salt bridge has been shown to stabilize partially-folded spike conformations, and its common occurrence here likely relates to the reduced ability of apo-form spikes to recover helicity.

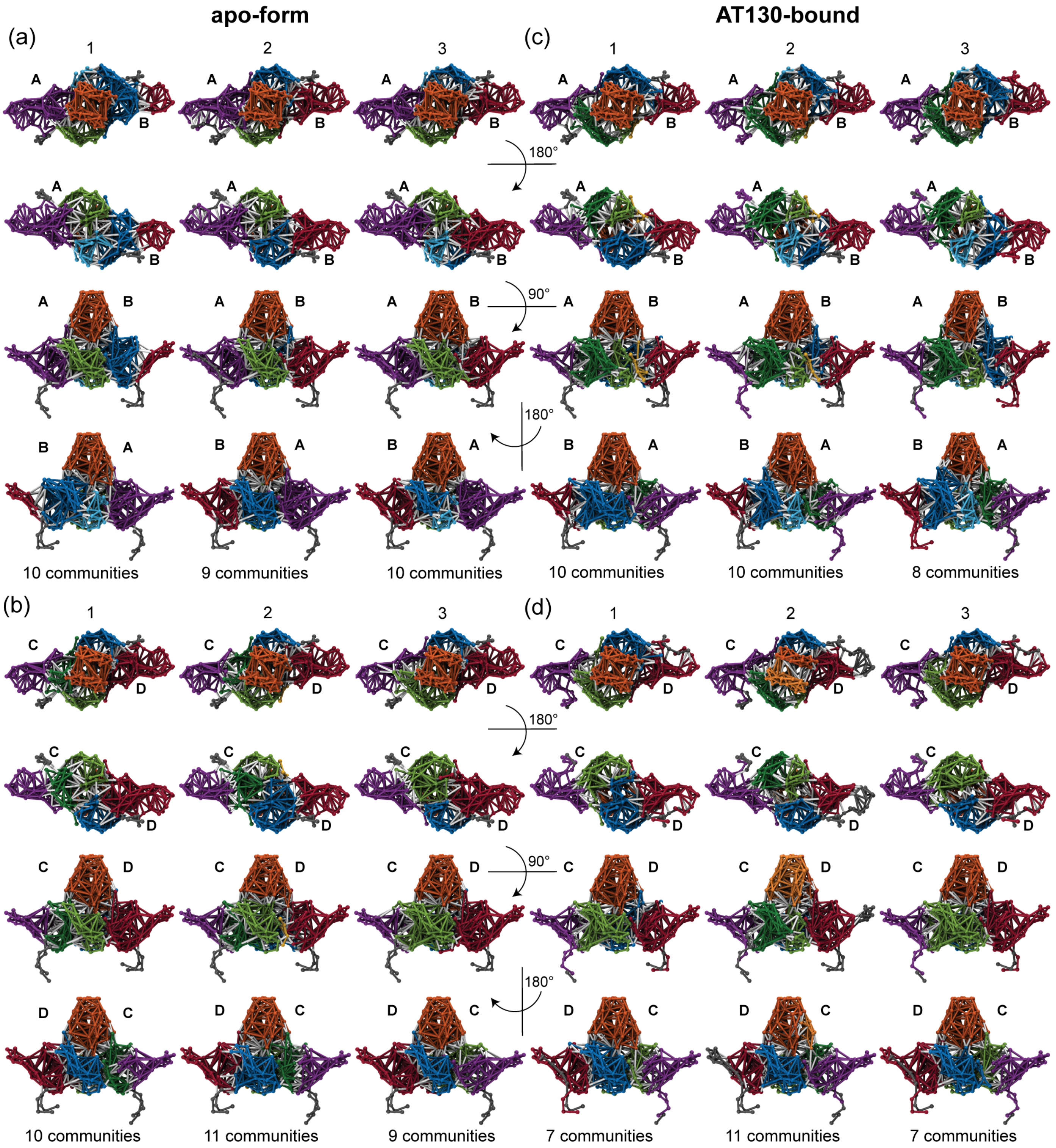

Figure S2. Complete set of twelve network models. Dynamical network models calculated in triplicate for apo-form (**a-b**) and AT130-bound (**c-d**) dimers. Each model based on an ensemble of one million randomly-selected conformers collected over 20 μs of cumulative sampling. Colors denote sub-network communities. Five major communities encompass helix 1,5 of each half-dimer (purple and red), where helix 5 forms the interdimer interface, helix 2-3a of each half-dimer (blue and green), which are part of the chassis sub-domain, and helix 3b-4a of both half dimers (orange), called the spike tip. The remainder of the fulcrum sub-domain participated in either the helix 1,5 or helix 2-3a community within the same half-dimer. Significant differences between network models involved the fragmentation of these five major communities, particularly 2-3a and 3b-4a, denoted by light and dark variations on each color (light and dark blue, green, and orange). A small community in helix 1 was also occasionally observed (yellow). Communities located in C-terminal tails shown in gray.

Table 1: Characteristics of hinge angle distributions, given as mean and standard deviation.

	apo-form	AT130-bound
Chain A	40.6 ± 8.01	38.4 ± 8.91
Chain B	37.0 ± 6.73	36.7 ± 8.25
Chain C	38.7 ± 7.43	35.7 ± 7.62
Chain D	39.3 ± 7.32	38.2 ± 9.42

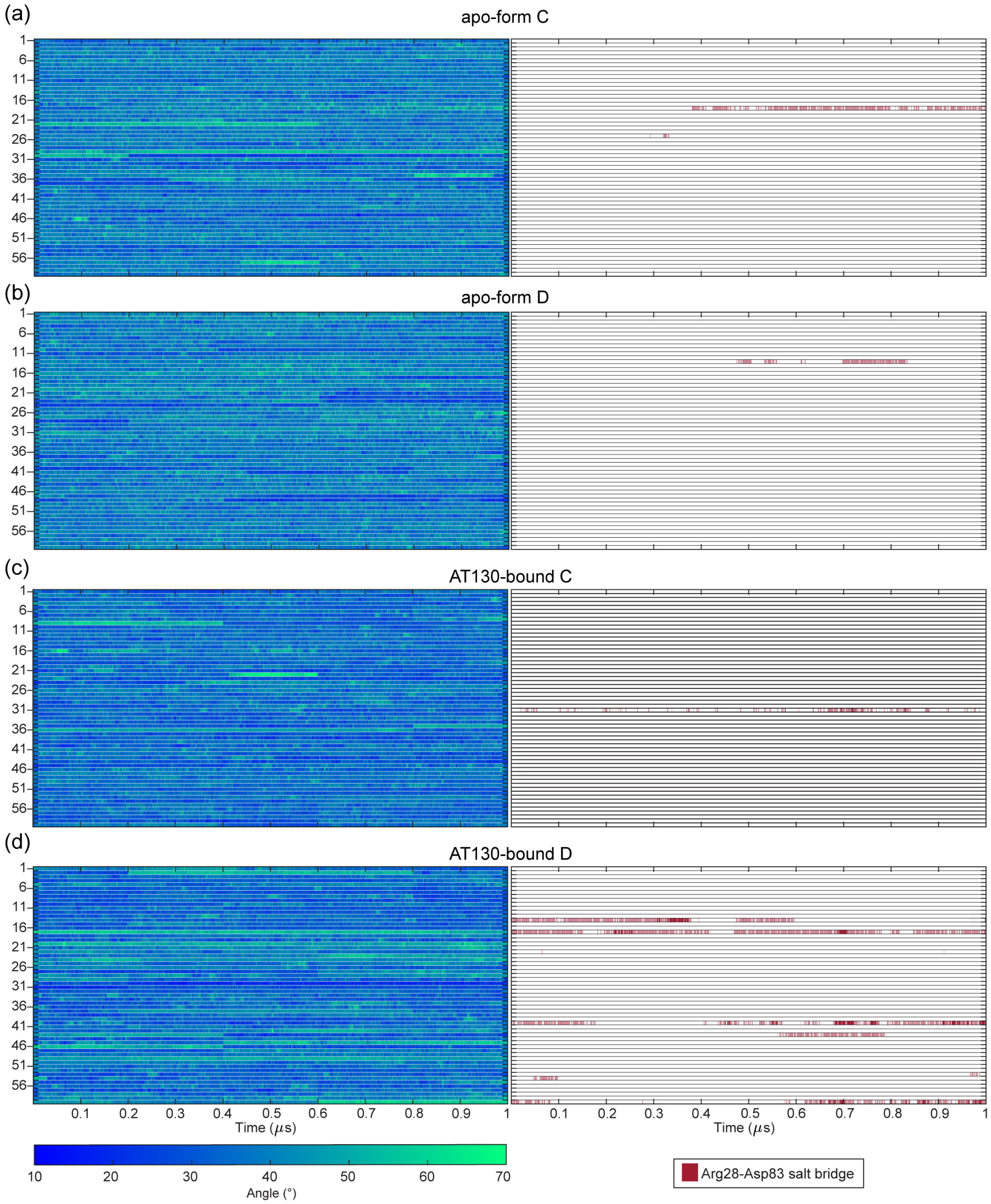

Figure S3. Hinge angle and Arg28–Asp83 salt bridge. Heat maps showing time series of hinge angle (left) and presence of Arg28–Asp83 salt bridge (right) for apo-form (**a-b**) and AT130-bound (**c-d**) CD dimers. Salt bridge interaction is typically correlated with dramatic curvature along helix 4, facilitating residue contact between the spike tip and fulcrum.

(a)

	Distance (Å) apo-form	Distance (Å) AT130-bound
Chain A	17.4 ± 2.8	18.0 ± 2.8
Chain B	18.1 ± 1.3	18.0 ± 2.7
Chain C	17.5 ± 2.9	17.6 ± 3.1
Chain D	16.8 ± 2.7	15.9 ± 3.7

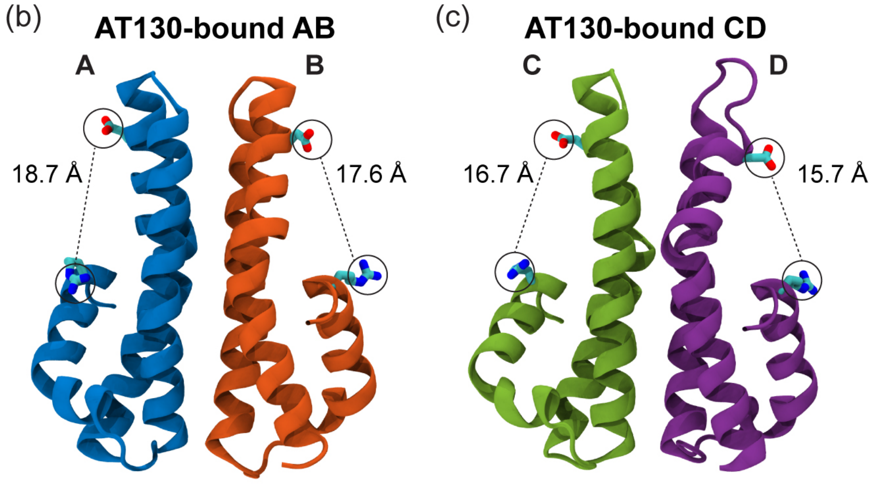

Figure S4. Distance separating Arg28–Asp83. (**a**) Mean distance and standard deviation characterizing the separation between Arg28–Asp83 for each quasi-equivalent chain in the apo-form and AT130-bound dimers. Representative conformation of the AT130-bound AB (**b**) and CD (**c**) dimer where the rare Arg28–Asp83 salt bridge is not intact; note that separation distances are near the mean. The mean values for C and particularly D chains are lower due to their increased likelihood to adopt open conformations that allow formation of the salt bridge. Distances were measured between centers of heavy atoms in the Arg28 and Asp83 functional groups.

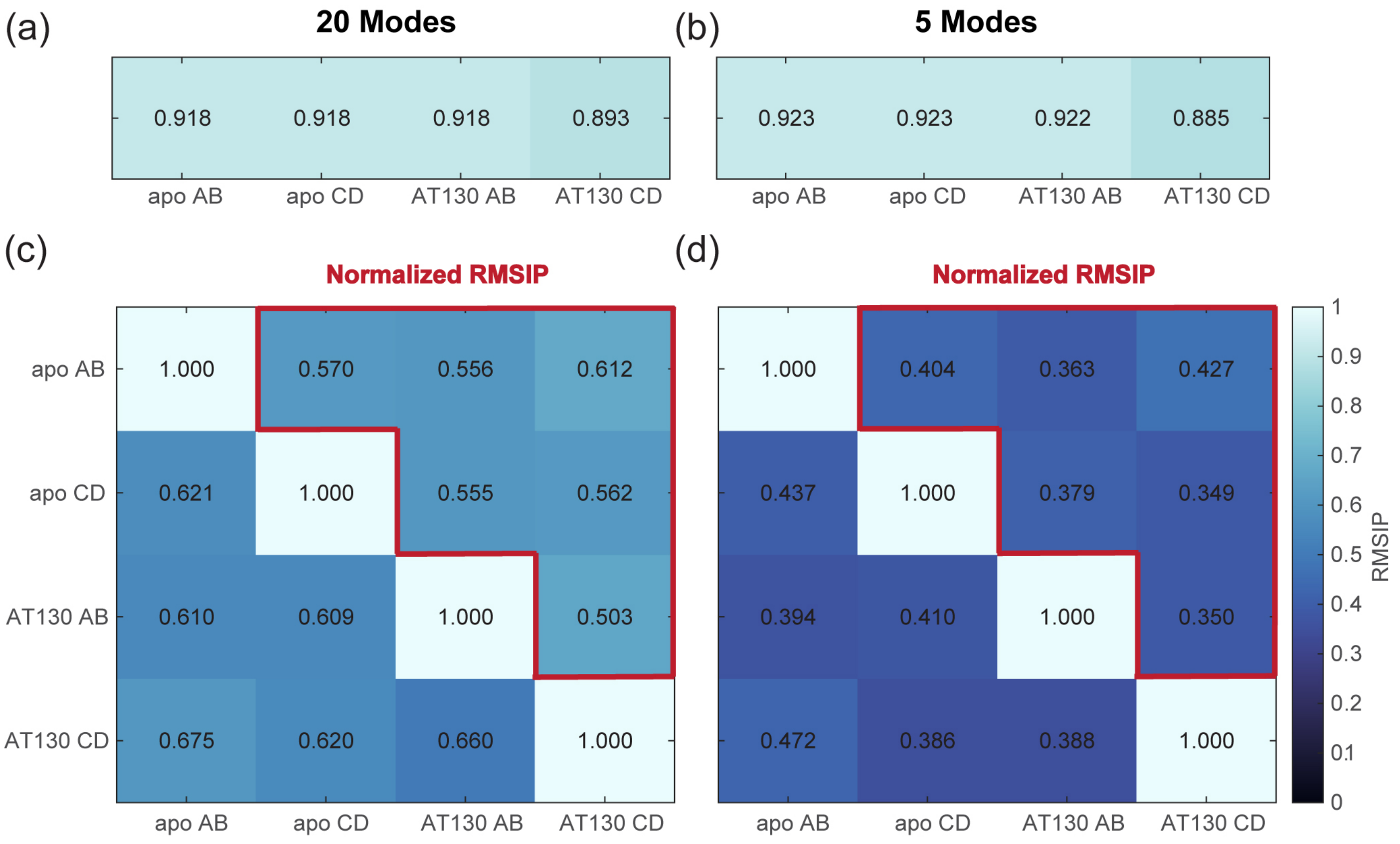

Figure S5. Root mean square inner product (RMSIP) analysis. RMSIP between first and second halves of simulation trajectories based on (**a**) 20 modes and (**b**) the top five modes from principal component analysis. RMSIP between each simulation system based on (**c**) 20 modes and (**d**) the top five modes. Values from panel (**a-b**) were used to normalize the results shown above the diagonal, highlighted in red. RMSIP scores range from zero (black) to one (light blue) indicating increasing overlap in the sampled mode sub-space. Values along the diagonal are one, reflecting that comparison of a sub-space with itself produces perfect overlap.
